# Supplementary material for: Cardiac Arrhythmia Risk after Anti-Cancer Drug Exposure and Related Disease Molecular Imaging Outlook: A Systematic Review, Meta-Analysis, and Network Meta-Analysis
Source: Biology (Basel). 2024 Jun 25;13(7):465. doi: 10.3390/biology13070465 (PMC11273816; doi:10.3390/biology13070465)
Supplement: Supplementary file 1 [file biology-13-00465-s001.zip › sup-figures and charts.pdf]

Table S1: Search Strategy

2022-3-18**Search Strategy-Pubmed**

#4	•••	>	Search: **"randomized controlled trial"[pt] OR "controlled clinical trial"[pt] OR "randomized"[tiab] OR "placebo"[tiab] OR "drug therapy"[sh] OR "randomly"[tiab] OR "trial"[tiab] OR "groups"[tiab] OR "cohort studies"[mesh] OR "case-control studies"[mesh] OR "comparative study"[pt] OR "risk factors"[mesh] OR "cohort"[tw] OR "compared"[tw] OR "groups"[tw] OR "case control"[tw] OR "multivariate"[tw] OR "case series"**	10,846,321	10:23:36
#3	•••	>	Search: **"humans"[MeSH Terms]**	20,269,290	10:21:33
#2	•••	>	Search: **"tachycardia, ventricular"[MeSH Terms] OR ("tachycardia"[All Fields] AND "ventricular"[All Fields]) OR "ventricular tachycardia"[All Fields] OR ("ventricular"[All Fields] AND "tachyarrhythmia"[All Fields]) OR "ventricular tachyarrhythmia"[All Fields] OR "arrhythmias, cardiac"[MeSH Terms] OR ("arrhythmias"[All Fields] AND "cardiac"[All Fields]) OR "cardiac arrhythmias"[All Fields] OR "arrhythmia"[All Fields] OR "torsades de pointes"[MeSH Terms] OR ("torsades"[All Fields] AND "de"[All Fields] AND "pointes"[All Fields]) OR "torsades de pointes"[All Fields]**	278,216	10:21:24
#1	•••	>	Search: **"Anthracyclines"[Mesh] OR Anthracyclin* OR Aclarubicin OR aclacinomycin OR Aldoxorubicin OR Annamycin OR Daunorubicin OR Cerubidine OR Doxorubicin OR Adriamycin OR Epirubicin OR "GPX-150" OR Idarubicin OR Pirarubicin OR Plicamycin OR Mithracin OR Sabarubicin OR SP1049C OR Valrubicin OR Valstar OR "Zoptarelin doxorubicin" OR Zorubicin OR "zorubicin" [Supplementary Concept] OR "LHRH, lysine(6)-doxorubicin" [Supplementary Concept] OR "valrubicin" [Supplementary Concept] OR "SP 1049C" [Supplementary Concept] OR "sabarubicin" [Supplementary Concept] OR "Plicamycin"[Mesh] OR "pirarubicin" [Supplementary Concept] OR "Idarubicin"[Mesh] OR "5-imino-13-deoxydoxorubicin" [Supplementary Concept] OR "Epirubicin"[Mesh] OR "Doxorubicin" [Mesh] OR "Daunorubicin"[Mesh] OR "annamycin" [Supplementary Concept] OR "DOXO-EMCH" [Supplementary Concept] OR "Aclarubicin"[Mesh]**	106,973	10:18:40

History and Search Details Download Delete

Search	Actions	Details	Query	Results	Time
#5	•••	>	Search: **((("Anthracyclines"[Mesh] OR Anthracyclin* OR Aclarubicin OR aclacinomycin OR Aldoxorubicin OR Annamycin OR Daunorubicin OR Cerubidine OR Doxorubicin OR Adriamycin OR Epirubicin OR "GPX-150" OR Idarubicin OR Pirarubicin OR Plicamycin OR Mithracin OR Sabarubicin OR SP1049C OR Valrubicin OR Valstar OR "Zoptarelin doxorubicin" OR Zorubicin OR "zorubicin" [Supplementary Concept] OR "LHRH, lysine(6)-doxorubicin" [Supplementary Concept] OR "valrubicin" [Supplementary Concept] OR "SP 1049C" [Supplementary Concept] OR "sabarubicin" [Supplementary Concept] OR "Plicamycin"[Mesh] OR "pirarubicin" [Supplementary Concept] OR "Idarubicin"[Mesh] OR "5-imino-13-deoxydoxorubicin" [Supplementary Concept] OR "Epirubicin"[Mesh] OR "Doxorubicin" [Mesh] OR "Daunorubicin"[Mesh] OR "annamycin" [Supplementary Concept] OR "DOXO-EMCH" [Supplementary Concept] OR "Aclarubicin"[Mesh]) AND ("tachycardia, ventricular"[MeSH Terms] OR ("tachycardia"[All Fields] AND "ventricular"[All Fields]) OR "ventricular tachycardia"[All Fields] OR ("ventricular"[All Fields] AND "tachyarrhythmia"[All Fields]) OR "ventricular tachyarrhythmia"[All Fields] OR "arrhythmias, cardiac"[MeSH Terms] OR ("arrhythmias"[All Fields] AND "cardiac"[All Fields]) OR "cardiac arrhythmias"[All Fields] OR "arrhythmia"[All Fields] OR "torsades de pointes"[MeSH Terms] OR ("torsades"[All Fields] AND "de"[All Fields] AND "pointes"[All Fields]) OR "torsades de pointes"[All Fields])) AND ("humans"[MeSH Terms])) AND ("randomized controlled trial"[pt] OR "controlled clinical trial"[pt] OR "randomized"[tiab] OR "placebo"[tiab] OR "drug therapy"[sh] OR "randomly"[tiab] OR "trial"[tiab] OR "groups"[tiab] OR "cohort studies"[mesh] OR "case-control studies"[mesh] OR "comparative study"[pt] OR "risk factors"[mesh] OR "cohort"[tw] OR "compared"[tw] OR "groups"[tw] OR "case control"[tw] OR "multivariate"[tw] OR "case series")**	379	10:23:58

((("Anthracyclines"[Mesh] OR Anthracyclin* OR Aclarubicin OR aclacinomycin OR Aldoxorubicin OR

Annamycin OR Daunorubicin OR Cerubidine OR Doxorubicin OR Adriamycin OR Epirubicin OR "GPX-150" OR Idarubicin OR Pirarubicin OR Plicamycin OR Mithracin OR Sabarubicin OR SP1049C OR Valrubicin OR Valstar OR "Zoptarelin doxorubicin" OR Zorubicin OR "zorubicin" [Supplementary Concept] OR "LHRH, lysine(6)-doxorubicin" [Supplementary Concept] OR "valrubicin" [Supplementary Concept] OR "SP 1049C" [Supplementary Concept] OR "sabarubicin" [Supplementary Concept] OR "Plicamycin"[Mesh] OR "pirarubicin" [Supplementary Concept] OR "Idarubicin"[Mesh] OR "5-imino-13-deoxydoxorubicin" [Supplementary Concept] OR "Epirubicin"[Mesh] OR "Doxorubicin"[Mesh] OR "Daunorubicin"[Mesh] OR "annamycin" [Supplementary Concept] OR "DOXO-EMCH" [Supplementary Concept] OR "Aclarubicin"[Mesh]) AND ("tachycardia, ventricular"[MeSH Terms] OR ("tachycardia"[All Fields] AND "ventricular"[All Fields]) OR "ventricular tachycardia"[All Fields] OR ("ventricular"[All Fields] AND "tachyarrhythmia"[All Fields]) OR "ventricular tachyarrhythmia"[All Fields] OR "arrhythmias, cardiac"[MeSH Terms] OR ("arrhythmias"[All Fields] AND "cardiac"[All Fields]) OR "cardiac arrhythmias"[All Fields] OR "arrhythmia"[All Fields] OR "torsades de pointes"[MeSH Terms] OR ("torsades"[All Fields] AND "de"[All Fields] AND "pointes"[All Fields]) OR "torsades de pointes"[All Fields])) AND ("humans"[MeSH Terms])) AND ("randomized controlled trial"[pt] OR "controlled clinical trial"[pt] OR "randomized"[tiab] OR "placebo"[tiab] OR "drug therapy"[sh] OR "randomly"[tiab] OR "trial"[tiab] OR "groups"[tiab] OR "cohort studies"[mesh] OR "case-control studies"[mesh] OR "comparative study"[pt] OR "risk factors"[mesh] OR "cohort"[tw] OR "compared"[tw] OR "groups"[tw] OR "case control"[tw] OR "multivariate"[tw] OR "case series")

1-Anthracycline

"Anthracyclines"[Mesh] OR Anthracyclin* OR Aclarubicin OR aclacinomycin OR Aldoxorubicin OR Annamycin OR Daunorubicin OR Cerubidine OR Doxorubicin OR Adriamycin OR Epirubicin OR "GPX-150" OR Idarubicin OR Pirarubicin OR Plicamycin OR Mithracin OR Sabarubicin OR SP1049C OR Valrubicin OR Valstar OR "Zoptarelin doxorubicin" OR Zorubicin OR "zorubicin" [Supplementary Concept] OR "LHRH, lysine(6)-doxorubicin" [Supplementary Concept] OR "valrubicin" [Supplementary Concept] OR "SP 1049C" [Supplementary Concept] OR "sabarubicin" [Supplementary Concept] OR "Plicamycin"[Mesh] OR "pirarubicin" [Supplementary Concept] OR "Idarubicin"[Mesh] OR "5-imino-13-deoxydoxorubicin" [Supplementary Concept] OR "Epirubicin"[Mesh] OR "Doxorubicin"[Mesh] OR "Daunorubicin"[Mesh] OR "annamycin" [Supplementary Concept] OR "DOXO-EMCH" [Supplementary Concept] OR "Aclarubicin"[Mesh]

2-arrhythmias

"tachycardia, ventricular"[MeSH Terms] OR ("tachycardia"[All Fields] AND "ventricular"[All Fields]) OR "ventricular tachycardia"[All Fields] OR ("ventricular"[All Fields] AND "tachyarrhythmia"[All Fields]) OR "ventricular tachyarrhythmia"[All Fields] OR "arrhythmias, cardiac"[MeSH Terms] OR ("arrhythmias"[All Fields] AND "cardiac"[All Fields]) OR "cardiac arrhythmias"[All Fields] OR "arrhythmia"[All Fields] OR "torsades de pointes"[MeSH Terms] OR ("torsades"[All Fields] AND "de"[All Fields] AND "pointes"[All Fields]) OR "torsades de pointes"[All Fields]

3-human

"humans"[MeSH Terms]

4-clinical trials

"randomized controlled trial"[pt] OR "controlled clinical trial"[pt] OR "randomized"[tiab] OR "placebo"[tiab] OR "drug therapy"[sh] OR "randomly"[tiab] OR "trial"[tiab] OR "groups"[tiab] OR "cohort studies"[mesh] OR

"case-control studies"[mesh] OR "comparative study"[pt] OR "risk factors"[mesh] OR "cohort"[tw] OR "compared"[tw] OR "groups"[tw] OR "case control"[tw] OR "multivariate"[tw] OR "case series"

Search Strategy-EMBASE-345

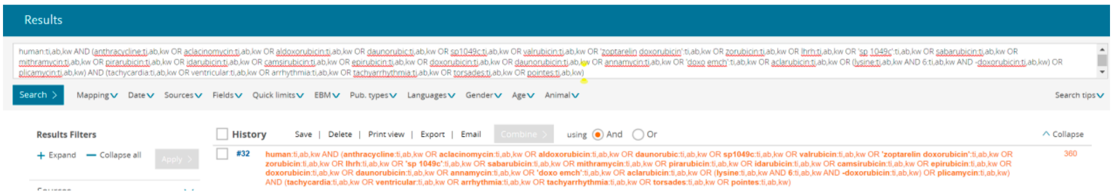

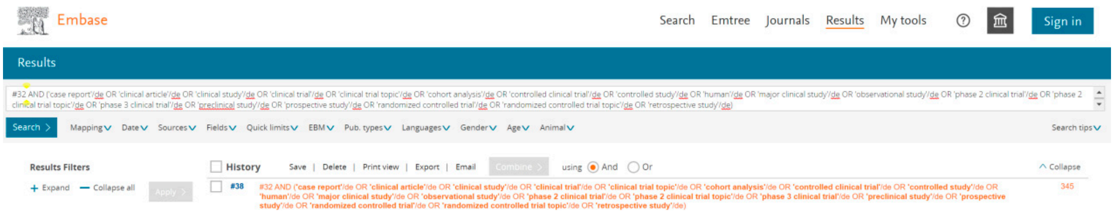

#1 human:ti,ab,kw
#2 anthracycline:ti,ab,kw OR aclacinomycin:ti,ab,kw OR aldoxorubicin:ti,ab,kw OR daunorubic:ti,ab,kw OR sp1049c:ti,ab,kw OR valrubicin:ti,ab,kw OR 'zoptarelin doxorubicin':ti,ab,kw OR zorubicin:ti,ab,kw OR lhrh:ti,ab,kw OR 'sp 1049c':ti,ab,kw OR sabarubicin:ti,ab,kw OR mithramycin:ti,ab,kw OR pirarubicin:ti,ab,kw OR idarubicin:ti,ab,kw OR camsirubicin:ti,ab,kw OR epirubicin:ti,ab,kw OR doxorubicin:ti,ab,kw OR daunorubicin:ti,ab,kw OR annamycin:ti,ab,kw OR 'doxo emch':ti,ab,kw OR aclarubicin:ti,ab,kw OR (lysine:ti,ab,kw AND 6:ti,ab,kw AND -doxorubicin:ti,ab,kw) OR plicamycin:ti,ab,kw
#3 tachycardia:ti,ab,kw OR ventricular:ti,ab,kw OR arrhythmia:ti,ab,kw OR tachyarrhythmia:ti,ab,kw OR torsades:ti,ab,kw OR pointes:ti,ab,kw
#4 'case report'/de OR 'clinical article'/de OR 'clinical study'/de OR 'clinical trial'/de OR 'clinical trial topic'/de OR 'cohort analysis'/de OR 'controlled clinical trial'/de OR 'controlled study'/de OR 'human'/de OR 'major clinical study'/de OR 'observational study'/de OR 'phase 2 clinical trial'/de OR 'phase 2 clinical trial topic'/de OR 'phase 3 clinical trial'/de OR 'preclinical study'/de OR 'prospective study'/de OR 'randomized controlled trial'/de OR 'randomized controlled trial topic'/de OR 'retrospective study'/de
#5 #1 AND #2 AND #3 AND #4

Search Strategy-Web of Science-263

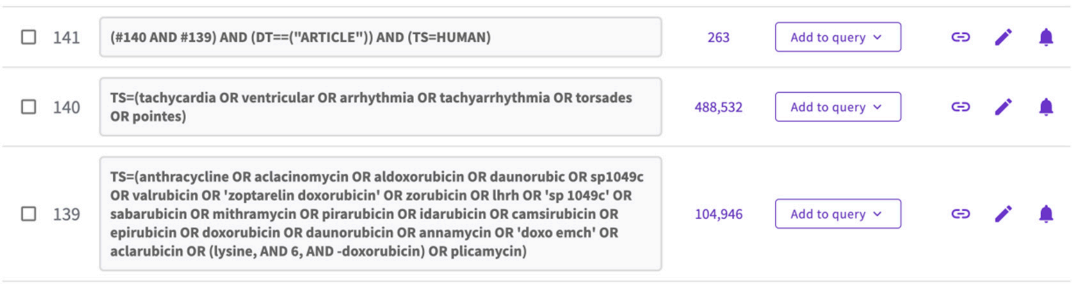

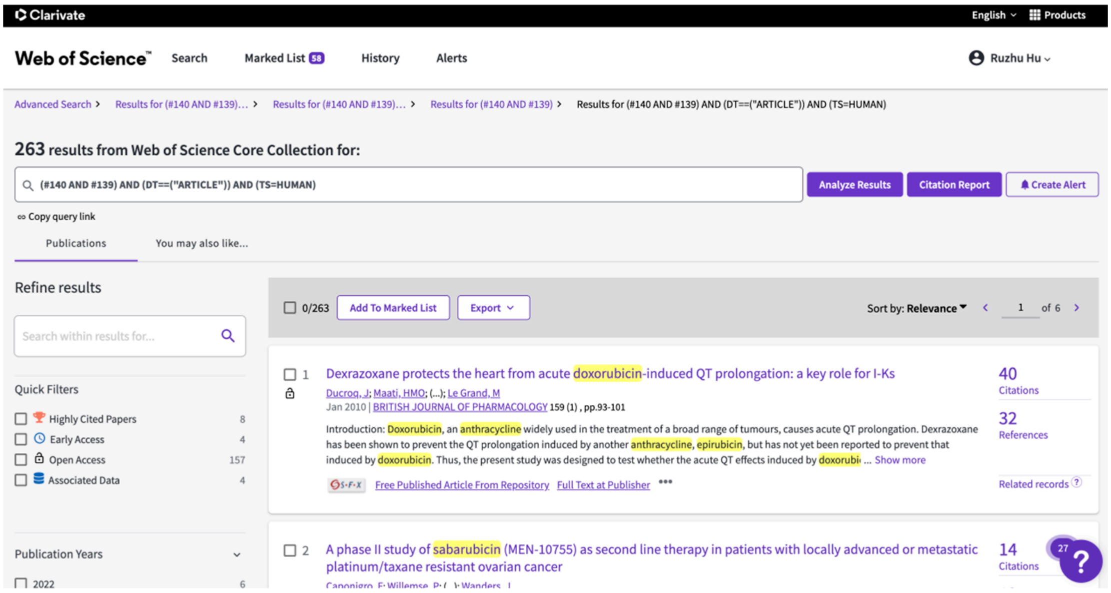

#1 TS=(anthracycline OR aclacinomycin OR aldoxorubicin OR daunorubic OR sp1049c OR valrubicin OR 'zoptarelin doxorubicin' OR zorubicin OR lhrh OR 'sp 1049c' OR sabarubicin OR mithramycin OR pirarubicin OR idarubicin OR camsirubicin OR epirubicin OR doxorubicin OR daunorubicin OR annamycin OR 'doxo emch' OR aclarubicin OR (lysine, AND 6, AND -doxorubicin) OR plicamycin)

#2 TS=(tachycardia OR ventricular OR arrhythmia OR tachyarrhythmia OR torsades OR pointes)

#3 #1 AND #2 and (DT=("ARTICLE")) AND Human

Search Strategy-Cochrane Library-247

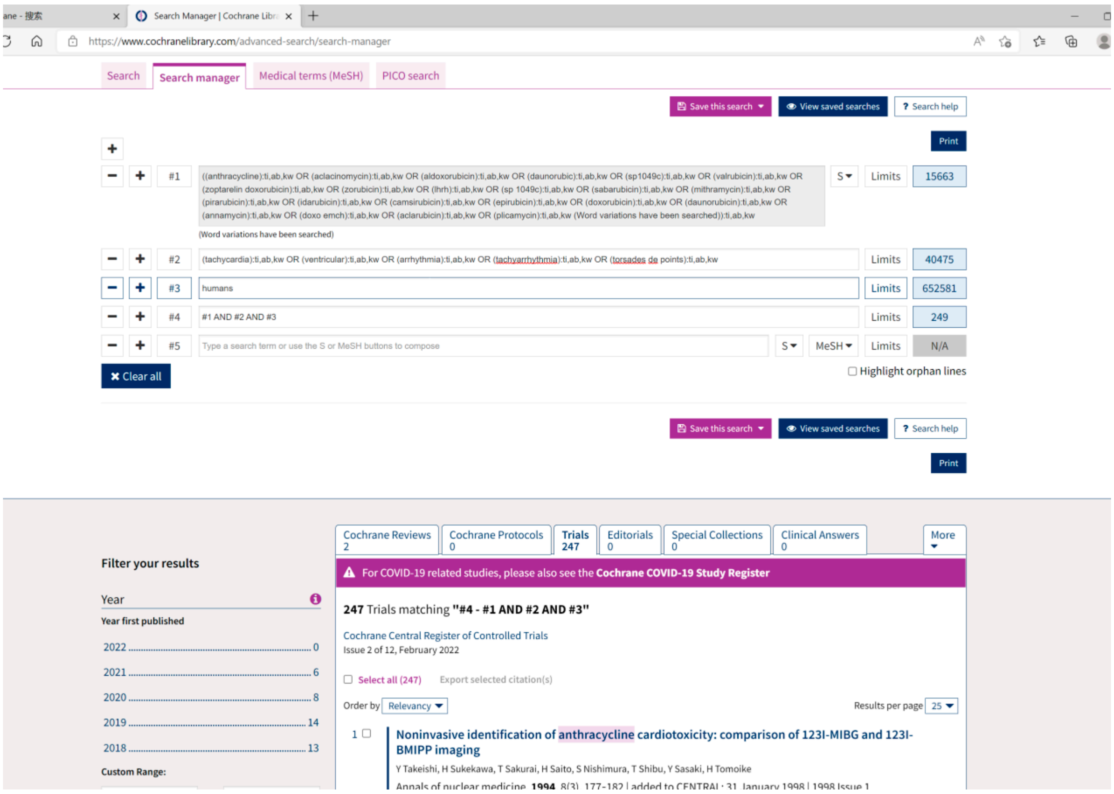

1# (anthracycline):ti,ab,kw OR (aclacinomycin):ti,ab,kw OR (aldoxorubicin):ti,ab,kw OR (daunorubic):ti,ab,kw OR (sp1049c):ti,ab,kw OR (valrubicin):ti,ab,kw OR (zoptarelin doxorubicin):ti,ab,kw OR (zorubicin):ti,ab,kw OR (lhrh):ti,ab,kw OR (sp 1049c):ti,ab,kw OR (sabarubicin):ti,ab,kw OR (mithramycin):ti,ab,kw OR (pirarubicin):ti,ab,kw OR (idarubicin):ti,ab,kw OR (camsirubicin):ti,ab,kw OR (epirubicin):ti,ab,kw OR (doxorubicin):ti,ab,kw OR (daunorubicin):ti,ab,kw OR (annamycin):ti,ab,kw OR (doxo emch):ti,ab,kw OR (aclarubicin):ti,ab,kw OR (plicamycin):ti,ab,kw (Word variations have been searched)

2# (tachycardia):ti,ab,kw OR (ventricular):ti,ab,kw OR (arrhythmia):ti,ab,kw OR (tachyarrhythmia):ti,ab,kw OR (torsades de points):ti,ab,kw

#3 humans

#4 #1 AND #2 AND #3

```
Tests for Publication Bias

Begg's Test

  adj. Kendall's Score (P-Q) =        6
          Std. Dev. of Score =     9.59
           Number of Studies =        9
                          z  =     0.63
                    Pr > |z| =    0.532
                          z  =     0.52 (continuity corrected)
                    Pr > |z| =    0.602 (continuity corrected)

Egger's test
```

Std_Eff	Coef.	Std. Err.	t	P>|t|	[95% Conf.	Interval]
slope	.5305742	.0704218	7.53	0.000	.3640531	.6970952
bias	.686831	.2829191	2.43	0.046	.0178335	1.355829

Figure S1: Tests for publication bias for Arrhythmia in anthracycline users versus non-users

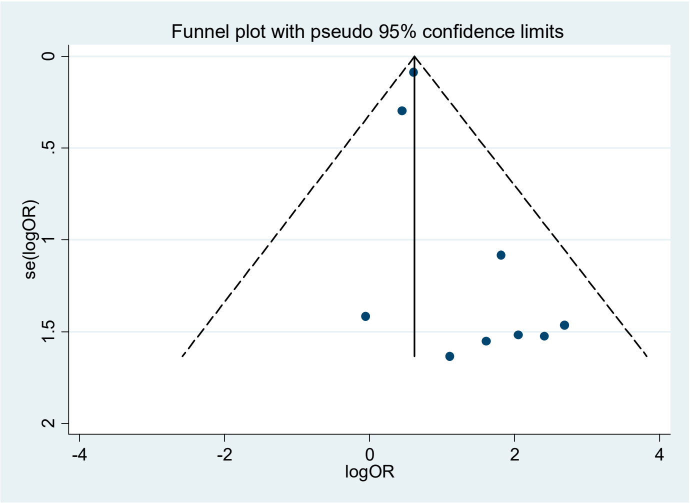

Figure S2: Funnel plot for publication bias for Arrhythmia in anthracycline users versus non-users

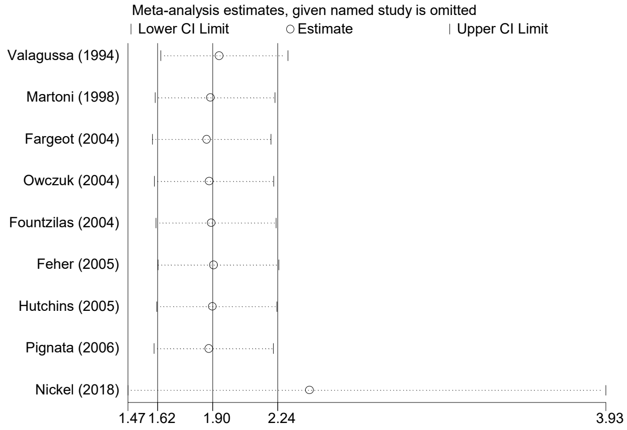

Figure S3: Sensitive analysis for Arrhythmia in anthracycline users versus non-users

```
Meta-regression                                      Number of obs  =        9
REML estimate of between-study variance              tau2           =        0
% residual variation due to heterogeneity            I-squared_res  =    0.00%
Proportion of between-study variance explained       Adj R-squared  =       .%
With Knapp-Hartung modification
```

logOR	Coef.	Std. Err.	t	P>|t|	[95% Conf.	Interval]
var13	-.9601124	.5796465	-1.66	0.142	-2.330759	.4105337
_cons	2.523645	1.150159	2.19	0.064	-.1960497	5.24334

```
. metareg logOR year, wsse(_selogES) bsest(reml)

Meta-regression                                      Number of obs  =        9
REML estimate of between-study variance              tau2           =     .352
% residual variation due to heterogeneity            I-squared_res  =    0.00%
Proportion of between-study variance explained       Adj R-squared  =       .%
With Knapp-Hartung modification
```

logOR	Coef.	Std. Err.	t	P>|t|	[95% Conf.	Interval]
year	-.0047688	.0363676	-0.13	0.899	-.0907644	.0812269
_cons	10.51823	72.95485	0.14	0.889	-161.9926	183.029

Figure S4: Regression analysis for Arrhythmia in anthracycline users versus non-users by study types and years

```
Tests for Publication Bias

Begg's Test

  adj. Kendall's Score (P-Q) =       1
          Std. Dev. of Score =    1.91
           Number of Studies =       3
                           z =    0.52
                    Pr > |z| =   0.602
                           z =    0.00 (continuity corrected)
                    Pr > |z| =   1.000 (continuity corrected)

Egger's test
```

Std_Eff	Coef.	Std. Err.	t	P>|t|	[95% Conf.	Interval]
slope	.2895531	.1104463	2.62	0.232	-1.113801	1.692907
bias	1.004464	.2052697	4.89	0.128	-1.603735	3.612663

Figure S5: Tests for Publication Bias for Supraventricular arrhythmia in anthracycline users versus non-users.

```
. metareg logOR var13, wsse(_selogES) bsest(reml)

Meta-regression                                      Number of obs  =        3
REML estimate of between-study variance              tau2           =        0
% residual variation due to heterogeneity            I-squared_res  =    0.00%
Proportion of between-study variance explained       Adj R-squared  =       .%
With Knapp-Hartung modification
```

logOR	Coef.	Std. Err.	t	P>|t|	[95% Conf.	Interval]
var13	-1.220245	1.133054	-1.08	0.476	-15.61706	13.17657
_cons	3.055383	2.195433	1.39	0.397	-24.84023	30.951

Figure S6: Regression analysis for Supraventricular arrhythmia in anthracycline users versus non-users via type of study.

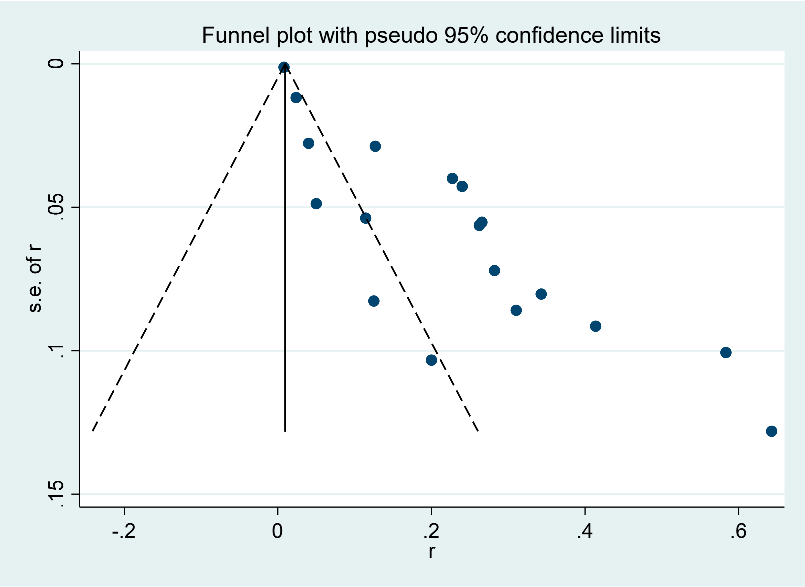

Figure S7: Funnel plot for publication bias for arrhythmia in anthracycline users in single-arm studies.

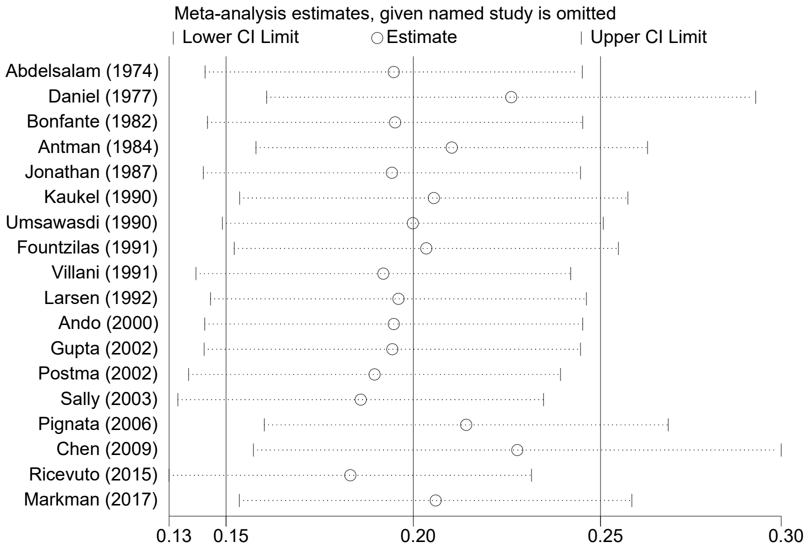

Figure S8: Sensitive analysis for arrhythmia in anthracycline users in single-arm studies.

```
Meta-regression                                      Number of obs  =        18
REML estimate of between-study variance              tau2           =    .02388
% residual variation due to heterogeneity            I-squared_res  =    92.25%
Proportion of between-study variance explained       Adj R-squared  =    -6.64%
With Knapp-Hartung modification
```

r	Coef.	Std. Err.	t	P>|t|	[95% Conf.	Interval]
year	.0027745	.0032976	0.84	0.413	-.0042161	.0097652
_cons	-5.317434	6.578516	-0.81	0.431	-19.26327	8.628396

Figure S9: Regression analysis via type of study for arrhythmia in anthracycline users in single-arm studies.

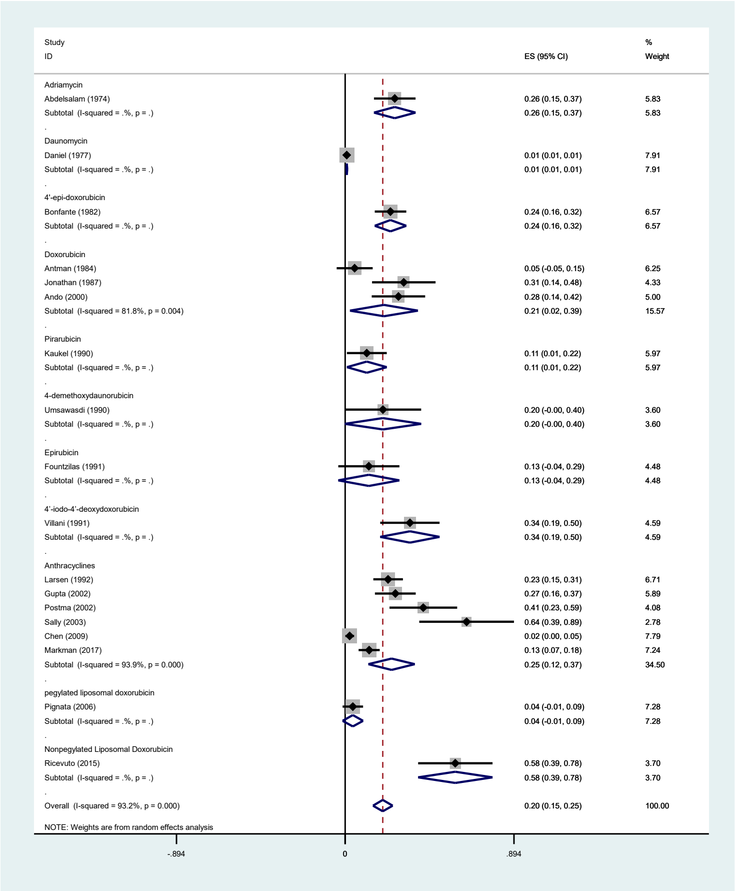

Figure S10: Subgroup analysis via type of anthracyclines for arrhythmia in single-arm studies.

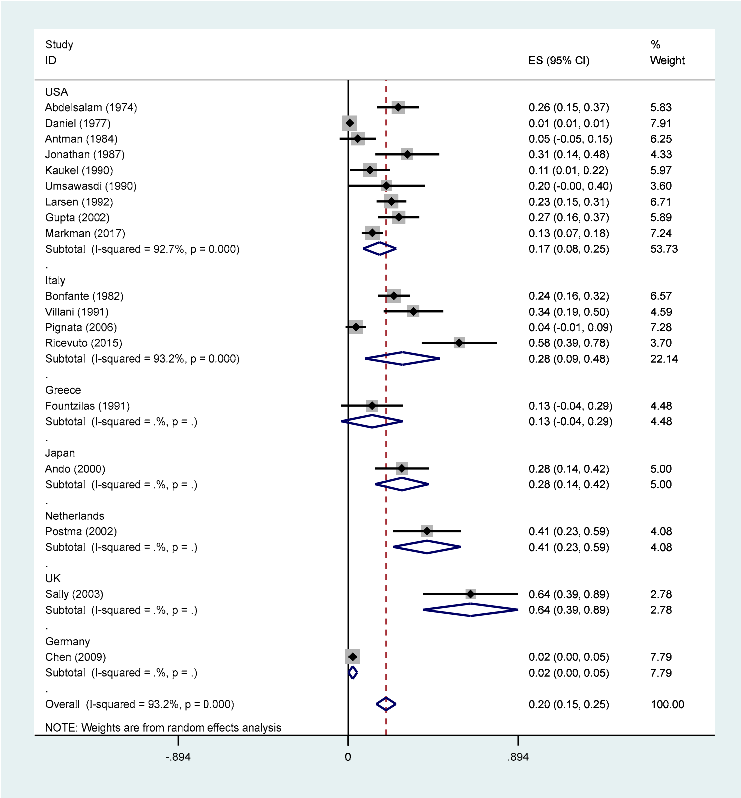

Figure S11: Subgroup analysis via countries for arrhythmia in single-arm studies.

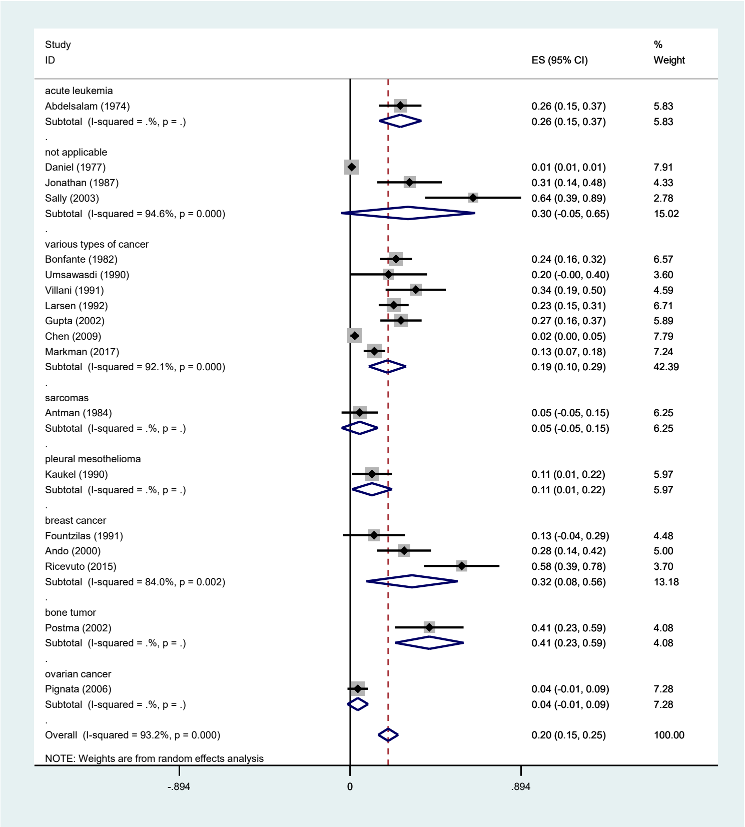

Figure S12: Subgroup analysis via different type of carcinomas for arrhythmia in single-arm studies.

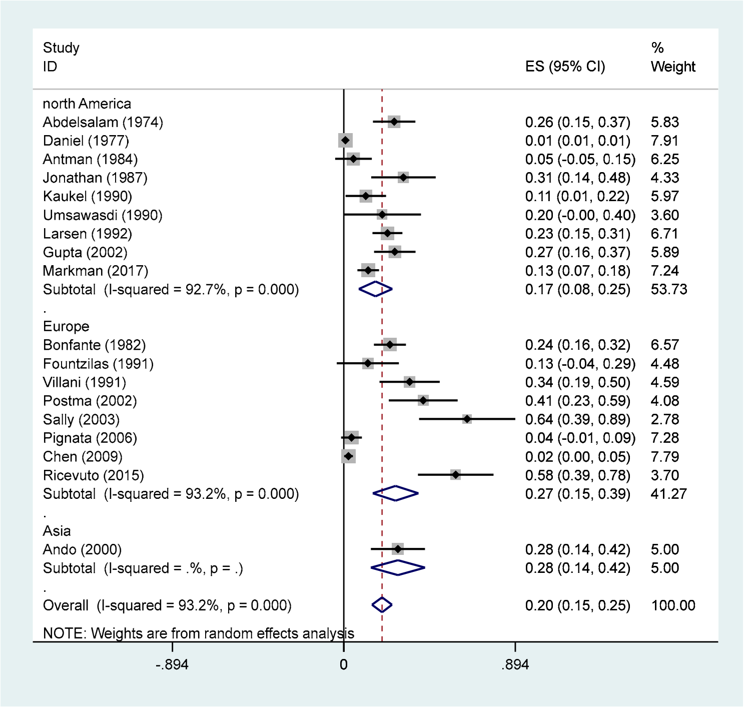

Figure S13: Subgroup analysis via continents for arrhythmia in single-arm studies.

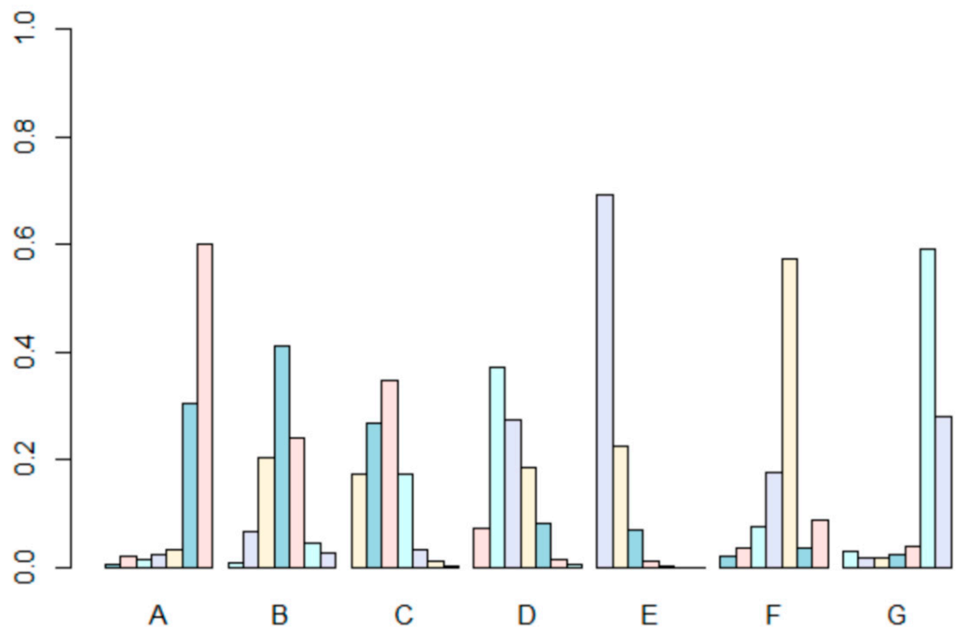

Figure S14:Treatment ranked by probability of highest risk of arrhythmia.

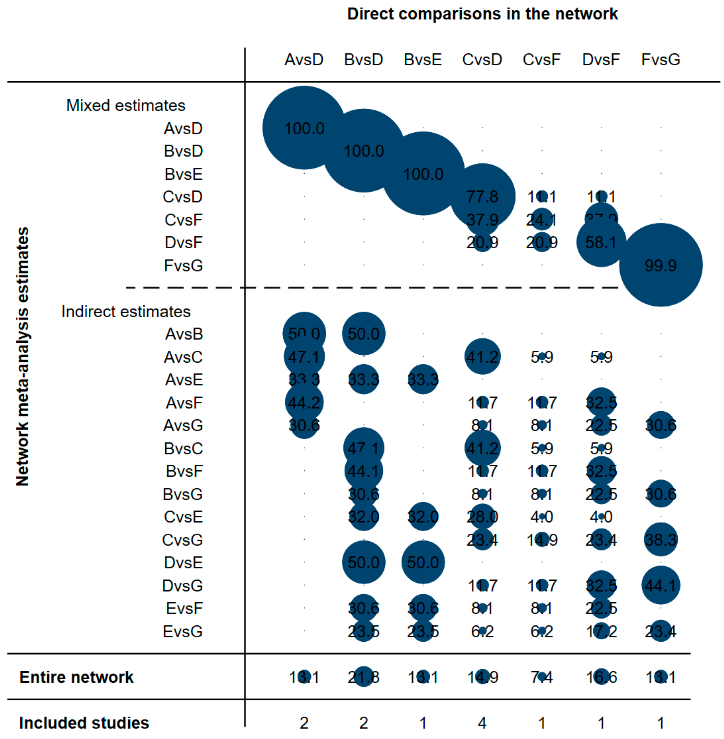

Figure S15: Direct comparisons in the network
