# Supplementary material for: Cardiac Arrhythmia Risk after Anti-Cancer Drug Exposure and Related Disease Molecular Imaging Outlook: A Systematic Review, Meta-Analysis, and Network Meta-Analysis
Source: Biology (Basel). 2024 Jun 25;13(7):465. doi: 10.3390/biology13070465 (PMC11273816; doi:10.3390/biology13070465)
Supplement: Supplementary file 1 [file biology-13-00465-s001.zip › sup-List of articles removed.pdf]

Supplementary Materials: List of articles removed

Number	Title	Reason	References
1	Aclarubicin-associated QTc prolongation and ventricular fibrillation	improper study type	Iwata N, Karasawa M, Omine M, Maekawa T, Suzuki T, Kawai Y. Aclarubicin-associated QTc prolongation and ventricular fibrillation. *Cancer Treat Rep*. 1984;68(3):527-529.
2	Acute arrhythmogenicity of first-dose chemotherapeutic agents in children	no anthracycline related drugs	
3	Acute cardiotoxicity of anthracyclines--analysis by using Holter ECG	Reports not retrieved	
4	Age characteristics of risk cardiovascular complications in women with left breast cancer with preserved ejection fraction at the stage of chemotherapy and radiotherapy	Reports not retrieved	
5	Ambulatory electrocardiographic evaluation of the heart in patients treated by megachemotherapy and bone marrow transplantation	no anthracycline related drugs	
6	Anthracycline induced cardiotoxicity in adult cancer patients: a prospective cohort study from a specialized oncology treatment centre in Uganda	data not available	
7	Anthracycline therapy can induce very early increase in QT dispersion and QTc prolongation	Reports not retrieved	
8	Anthracycline treatment, cardiovascular risk factors and the cumulative incidence of cardiovascular disease in a cohort of newly diagnosed lymphoma patients from the modern treatment	improper study type	

	era		
9	Anthracycline-induced acute myocarditis and ventricular fibrillation arrest	improper study type	
10	Assessment of anthracycline-induced cardiotoxicity with electrocardiography	Anthracyclines is not the only control	
11	Assessment of cardiac and pulmonary function in adult patients with Hodgkin's disease treated with ABVD or MOPP/ABVD plus adjuvant low-dose mediastinal irradiation	Anthracyclines in both group	
12	Atrial Function in Patients with Breast Cancer After Treatment with Anthracyclines	no arrythmia related outcomes	
13	Bradycardia due to anthracyclines	improper study type	
14	Cardiac arrhythmias in patients with small cell lung cancer and cardiac disease before, during and after doxorubicin administration. An evaluation of acute cardiotoxicity by continuous 24-hour Holter monitoring	no arrythmia related outcomes	
15	Cardiac complications associated with trastuzumab in the setting of adjuvant chemotherapy for breast cancer overexpressing human epidermal growth factor receptor type 2-a prospective study	no anthracycline related drugs	
16	Cardiac complications in patients undergoing a reduced-intensity conditioning hematopoietic stem cell transplantation	Anthracyclines in both group	
17	Cardiac effects of anthracycline treatment and their implications for aeromedical certification	Reports not retrieved	
18	Cardiac safety of trastuzumab emtansine (T-DM1) following anthracycline-based chemotherapy as (neo)adjuvant therapy for human epidermal growth factor receptor 2-positive (HER2+)	Anthracyclines in both group	

	early-stage breast cancer (EBC): Final data from TDM4874g		
19	Cardiac toxicity 4 to 20 years after completing anthracycline therapy	data not available	
20	Cardiac toxicity events in the PHARE trial, an adjuvant trastuzumab randomised phase III study	no anthracycline related drugs	
21	Cardiotoxicity of 5-fluorouracil in combination with folinic acid in patients with gastrointestinal cancer	no anthracycline related drugs	
22	Cardiotoxicity with trabectedin in the treatment of advanced soft tissue sarcoma	no anthracycline related drugs	
23	CHOP-rituximab with pegylated liposomal doxorubicin for the treatment of elderly patients with diffuse large B-cell lymphoma	no arrythmia related outcomes	
24	Clinical Application of the Heart Rate Deceleration Capacity Test to Predict Epirubicin-induced Cardiotoxicity	data not available	
25	Clinical studies on aclacinomycin A cardiotoxicity in adult patients with acute non lymphoblastic leukaemia	Reports not retrieved	
26	CT-P6 compared with reference trastuzumab for HER2-positive breast cancer: a randomised, double-blind, active-controlled, phase 3 equivalence trial	no anthracycline related drugs	
27	Dispersion of hyperenhancement in late gadolinium enhancement cardiovascular magnetic resonance measured with Moran's I is associated with a decrement in LVEF 6 months after cardiotoxic chemotherapy	no arrythmia related outcomes	
28	Docetaxel with epirubicin--investigations on cardiac safety	Anthracyclines is not the only control	
29	Early and late arrhythmogenic effects of doxorubicin	Anthracyclines is not the only control	

30	ECG changes in adriamycin therapy	Reports not retrieved	
31	Effect of epirubicin on the heart conduction system in patients with Hodgkin's disease	Reports not retrieved	
32	Effect of trastuzumab plus anthracycline on cardiac function in her2-positive breast cancer	Reports not retrieved	
33	Effects of first-dose doxorubicin on cardiac rhythm as evaluated by continuous 24-hour monitoring	preexisting diagnoses of arrhythmia	
34	Electrocardiogram analysis of adriamycin cardiotoxicity in 160 cases	Reports not retrieved	
35	Electrocardiographic changes following adriamycin treatment	preexisting diagnoses of arrhythmia	
36	Electrocardiographic changes in patients with acute leukoses treated with rubidomycin or adriamycin at the Internal Clinic A	Reports not retrieved	
37	Electrocardiographic characteristics of diffuse large B-cell lymphoma patients treated with anthracycline-based chemotherapy	preexisting diagnoses of arrhythmia	
38	Evaluating the Utility of Baseline Cardiac Function Screening in Early-Stage Breast Cancer Treatment	Anthracyclines is not the only control	
39	Evaluation of cardiotoxicity in new anthracycline analog ME 2303. ME 2303 Study Group	Reports not retrieved	
40	Is there a cardioprotective effect of angiotensin-converting enzyme inhibitor or beta blocker therapy in the treatment of breast cancer?	data not available	
41	Late cardiac effects of adjuvant chemotherapy in breast cancer	no arrythmia related	

	survivors treated on Southwest Oncology Group protocol s8897	outcomes	
42	The late cardiac sequelae after mantle-field irradiation. The results in Erlangen's patient caseload	Reports not retrieved	
43	Liver, gastrointestinal, and cardiac toxicity in intermediate hepatocellular carcinoma treated with PRECISION TACE with drug-eluting beads: results from the PRECISION V randomized trial	no arrythmia related outcomes	
44	Long-term efficacy analysis of the randomised, phase II TRYPHAENA cardiac safety study: evaluating pertuzumab and trastuzumab plus standard neoadjuvant anthracycline-containing and anthracycline-free chemotherapy regimens in patients with HER2-positive early breast cancer	Anthracyclines is not the only control	
45	Neoadjuvant chemotherapy with or without anthracyclines in the presence of dual HER2 blockade for HER2-positive breast cancer (TRAIN-2): a multicentre, open-label, randomised, phase 3 trial	Anthracyclines is not the only control	
46	Noninvasive identification of anthracycline cardiotoxicity: comparison of 123I-MIBG and 123I-BMIPP imaging	no arrythmia related outcomes	
47	Pegylated liposomal doxorubicin in combination with cyclophosphamide and trastuzumab in HER2-positive metastatic breast cancer patients: Efficacy and cardiac safety from the GEICAM/2004-05 study	no arrythmia related outcomes	
48	Phase I clinical and pharmacokinetic study of S9788, a new multidrug-resistance reversal agent given alone and in combination with doxorubicin to patients with advanced solid tumors	Anthracyclines is not the only control	
49	Phase IB study of doxorubicin in combination with the multidrug	Anthracyclines in both	

	resistance reversing agent S9788 in advanced colorectal and renal cell cancer	group	
50	A phase II study of high-dose epirubicin (EPI) plus cyclophosphamide (CPA) with G-CSF for breast cancer patients with visceral metastases or hormone-independent tumors: a trial of the Japan Clinical Oncology Group	Anthracyclines is not the only control	
51	Phase II study of high-dose epirubicin and etoposide in advanced non-small cell lung cancer	Anthracyclines is not the only control	
52	A phase II study of menogaril (7R-O-methylnogarol) in patients with relapsed/refractory acute myeloid leukemia: a study of the Eastern Cooperative Oncology Group	Reports not retrieved	
53	A phase II trial of epirubicin plus paclitaxel in metastatic breast cancer. United Kingdom Coordinating Committee for Cancer Research Breast Cancer Sub-Committee	Anthracyclines in both group	
54	A phase III randomized trial comparing adjuvant concomitant chemoradiotherapy versus standard adjuvant chemotherapy followed by radiotherapy in operable node-positive breast cancer: final results	data not available	
55	Phase III trial of liposomal doxorubicin and cyclophosphamide compared with epirubicin and cyclophosphamide as first-line therapy for metastatic breast cancer	Anthracyclines in both group	
56	Postmenopausal patients with node-positive resectable breast cancer: tamoxifen vs FEC 50 (6 cycles) vs FEC 50 (6 cycles) plus tamoxifen vs control - preliminary results of a 4-Arm randomised trial	Anthracyclines is not the only control	
57	Postoperative complications in patients of esophageal cancer after	Anthracyclines is not	

	neoadjuvant chemotherapy	the only control	
58	Prospective evaluation of paclitaxel versus combination chemotherapy with fluorouracil, doxorubicin, and cyclophosphamide as neoadjuvant therapy in patients with operable breast cancer	Anthracyclines is not the only control	
59	Protective role of black seed oil in doxorubicin-induced cardiac toxicity in children with acute lymphoblastic leukemia	Anthracyclines in both group	
60	QT dispersion correlates with systolic rather than diastolic parameters in patients receiving anthracycline treatment	data not available	
61	A randomized phase III study evaluating pegylated liposomal doxorubicin versus capecitabine as first-line therapy for metastatic breast cancer: results of the PELICAN study	data not available	
62	Randomized phase III trial of amrubicin/cisplatin versus etoposide/cisplatin as first-line treatment for extensive small-cell lung cancer	no arrythmia related outcomes	
63	Randomized trial comparing mitoxantrone with adriamycin in advanced breast cancer	Reports not retrieved	
64	A randomized trial of amsacrine and rubidazone in 39 patients with acute promyelocytic leukemia	Anthracyclines is not the only control	
65	Results of chemotherapy in thyroid cancer	Reports not retrieved	
66	Safety and efficacy of aerobic training in operable breast cancer patients receiving neoadjuvant chemotherapy: a phase II randomized trial	Anthracyclines in both group	
67	Safety and immunogenicity of neoadjuvant treatment using WT1-immunotherapeutic in combination with standard therapy in patients with WT1-positive Stage II/III breast cancer: a	no arrythmia related outcomes	

	randomized Phase I study		
68	Sequentially administered 5-azacitidine and amsacrine in refractory adult acute leukemia: a phase I-II trial of the Southeastern Cancer Study Group	Anthracyclines is not the only control	
69	Short term quality of life with epirubicin-fluorouracil-cyclophosphamid (FEC) and sequential epirubicin/cyclophosphamid-docetaxel (EC-DOC) chemotherapy in patients with primary breast cancer - Results from the prospective multi-center randomized ADEBAR trial	Anthracyclines is not the only control	
70	Signal-averaged ECG in patients after anthracycline therapy for childhood cancer	data not available	
71	Strain-Guided Management of Potentially Cardiotoxic Cancer Therapy	no arrythmia related outcomes	
72	Studies on adriamycin using a weekly regimen demonstrating its clinical effectiveness and lack of cardiac toxicity	data not available	
73	Subacute cardiotoxicity caused by anthracycline therapy in children: can dexrazoxane prevent this effect?	Reports not retrieved	
74	Superoxide dismutase activity in Adriamycin-induced cardiotoxicity in humans: A potential novel tool for risk stratification	no arrythmia related outcomes	
75	Systemic toxic effects associated with high-dose verapamil infusion and chemotherapy administration	Anthracyclines is not the only control	
76	Tamoxifen plus tegafur-uracil (TUFT) versus tamoxifen plus Adriamycin (doxorubicin) and cyclophosphamide (ACT) as adjuvant therapy to treat node-positive premenopausal breast	Anthracyclines is not the only control	

	cancer (PreMBC): results of Japan Clinical Oncology Group Study 9404		
77	Trastuzumab administered concurrently with anthracycline-containing adjuvant regimen for breast cancer	Reports not retrieved	
78	Trastuzumab as adjuvant therapy in HER21 early breast cancer - Cardiac safety analysis	data not available	
79	Trastuzumab Emtansine Plus Non-Pegylated Liposomal Doxorubicin in HER2-Positive Metastatic Breast Cancer (Thelma): A Single-Arm, Multicenter, Phase Ib Trial	Anthracyclines is not the only control	
80	Trastuzumab plus weekly epirubicin and paclitaxel for locally advanced and metastatic breast cancer: preliminary results of a feasibility-phase II study aimed at cardiotoxicity	Anthracyclines is not the only control	
81	Trastuzumab-associated cardiac adverse effects in the herceptin adjuvant trial	no arrythmia related outcomes	
82	Trastuzumab-associated cardiac events in the Persephone trial	Anthracyclines is not the only control	
83	Trastuzumab-related cardiac events in the treatment of early breast cancer	no arrythmia related outcomes	
84	Using biomarkers to detect the temporal trend of subclinical cardiotoxicity in patients with breast cancer treated with anthracyclines and her2+ antagonists	data not available	
85	Using health-related quality of life measures to predict cardiac function in survivors exposed to anthracyclines	Anthracyclines is not the only control	
86	Utilization of Cardiac Surveillance Tests in Survivors of Breast Cancer and Lymphoma After Anthracycline-Based Chemotherapy	preexisting diagnoses of arrhythmia	

87	Ventricular arrhythmia and torsade de pointe: dose limiting toxicities of the MDR-modulator S9788 in a phase I trial	data not available	
88	Ventricular repolarization time indexes following anthracycline treatment	data not available	
89	Weekly combination of non-pegylated liposomal doxorubicin and taxane in first-line breast cancer: wALT trial (phase I-II)	no arrythmia related outcomes	
90	Signal-averaged electrocardiography in survivors of Hodgkin's disease treated with and without dexrazoxane	data not available	
91	Preliminary evaluation of myocardial toxicity of 4'-deoxydoxorubicin: experimental and clinical results	data not available	
92	Evaluation of dexrazoxane effect on preventing acute cardiac arrhythmia in patients with breast cancer treated with neoadjuvant/adjuvant anthracycline-based chemotherapy	data not available	
