# Supplementary material for: Cardiac Arrhythmia Risk after Anti-Cancer Drug Exposure and Related Disease Molecular Imaging Outlook: A Systematic Review, Meta-Analysis, and Network Meta-Analysis
Source: Biology (Basel). 2024 Jun 25;13(7):465. doi: 10.3390/biology13070465 (PMC11273816; doi:10.3390/biology13070465)
Supplement: Supplementary file 1 [file biology-13-00465-s001.zip › sup-quality assessment.pdf]

Supplementary material: Quality Assessment according to Newcastle- Ottawa Quality Assessment Scale Cohort Studies

		**Valagussa1994**	**Owczuk2004**	**Nickel2018**	**Zhai2010**
**selection**	**representativeness of the exposed cohort**	1<br>turly representative of the average adjuvant chemotherapy with Dox in the community	1<br>turly representative of the average schedules for breast cancer surgery in the community	1<br>somewhat representative of the average anthracyclines vs non-anthracyclines users in the community	1<br>somewhat representative of the average non-Hodgkin's lymphoma in the community
	**selection of the non exposed cohort**	1<br>drawn from the same community as the exposed cohort	1<br>drawn from the same community as the exposed cohort	1<br>drawn from the same community as the exposed cohort	1<br>drawn from the same community as the exposed cohort
	**ascertainment of exposure**	1<br>structured interview	1<br>structured interview	0<br>written self report	0<br>no description
	**demonstration that outcome of interest was not present at start of study**	1<br>yes	1<br>yes	1<br>yes	1<br>yes
**comparability**	**comparability of cohorts on the basis of the design or analysis**	1<br>study controls for any additional factor	1<br>study controls for EPI/adriamycon	1<br>study controls for anthracyclines	1<br>study controls for pirarubin vs DOX
**outcome**	**assessment of outcome**	0<br>self report	0<br>self report	0<br>self report	0<br>self report
	**was follow-up long enough for outcomes to occur**	1<br>yes	0<br>no(33.1d)	1<br>yes(6m)	1<br>yes(5y)
	**adequacy of follow up of cohorts**	1<br>no description of the lost	1<br>complete follow up subjects accounted for	0<br>no description of the lost	1<br>complete follow up- all subjects accounted for
	**Total scores**	6	6	5	6

Supplementary material： Risk of Bias for Randomized Controlled Trials

Unique ID	Study ID	Reference	Experimental	Comparator	Outcome	Result	Aim	Effect of adhering to intervention?	Randomization process	Deviations from intended interventions	Missing outcome data	Measurement of the outcome	Selection of the reported result	Overall Bias
1	Martoni 1998	RCT	Epirubicin	Vinorelbine	arrhythmia	102/110	adhering to intervention (the 'per-protocol' effect)	occurance of non-protocol interventions	Some concerns	Low	Low	Low	Low	Some concerns
2	Fargeot 2004	RCT	Epirubicin+ tamoxifen	tamoxifen	arrhythmia	174/164	adhering to intervention (the 'per-protocol' effect)	occurance of non-protocol interventions	Some concerns	Low	Low	Some concerns	Low	Some concerns
3	Fountzilas2004	RCT	Epirubicin+paclitaxel	Paclitaxel+carboplatin	arrhythmia	162/160	adhering to intervention (the 'per-protocol' effect)	occurance of non-protocol interventions	Low	Low	Low	Low	Low	Low
4	Feher2005	RCT	Epirubicin	gemcitabine	arrhythmia	199/198	adhering to intervention (the 'per-protocol' effect)	failures in implementing the intervention that could have affected the outcome	Some concerns	Some concerns	Low	Low	Low	Some concerns
5	Hutchins2005	RCT	doxorubicin +cyclophosphamide + fluorouracil	methotrexate +cyclophosphamide + fluorouracil	arrhythmia	669/676	adhering to intervention (the 'per-protocol' effect)	failures in implementing the intervention that could have affected the outcome	Some concerns	Low	Low	Low	Low	Some concerns
6	Pignata 2011	RCT	pegylated liposomal doxorubicin	pegylated liposomal doxorubicin+carboplati	arrhythmi	403/40	adhering to intervention (the 'per-protocol' effect)	failures in implementing the intervention that could have affected	Low	Low	Low	Low	Low	Low

			30+carboplatin	n	a	8		the outcome						
7	Heidenreich2004	RCT	pegylated liposomal doxorubicin25	pegylated liposomal doxorubicin50	arrhythmia	22/26	assignment to intervention (the 'intention-to-treat' effect)	NA	Some concerns	Some concerns	Low	Low	Some concerns	Some concerns
8	Vici2011	RCT	Epirubicin	pegylated liposomal doxorubicin 40	arrhythmia	54/50	assignment to intervention (the 'intention-to-treat' effect)	NA	Low	Low	Low	Low	Low	Low

**Intention-to-treat**	**Unique ID**	**Study ID**	**Experimental**	**Comparator**	**Outcome**	**Weight**	**D1**	**D2**	**D3**	**D4**	**D5**	**Overall**
	7	Heidenreich2004	lated liposomal doxorubic	lated liposomal doxorubici	arrhythmia	1	!	!	+	+	!	!
	8	Vici2011	Epirubicin	lated liposomal doxorubici	arrhythmia	1	+	+	+	+	+	+

+ Low risk
! Some concerns
- High risk

**Per-protocol**	**Unique ID**	**Study ID**	**Experimental**	**Comparator**	**Outcome**	**Weight**	**D1**	**D2**	**D3**	**D4**	**D5**	**Overall**
	1	Martoni1998	Epirubicin	Vinorelbine	arrhythmia	1	!	+	+	+	+	!
	2	Fargeot2004	Epirubicin+ tamoxifen	tamoxifen	arrhythmia	1	!	+	+	!	+	!
	3	Fountzilas2004	Epirubicin+paclitaxel	Paclitaxel+carboplatin	arrhythmia	1	+	+	+	+	+	+
	4	Feher2005	Epirubicin	gemcitabine	arrhythmia	1	!	!	+	+	+	!
	5	Hutchins2005	doxorubicin +cyclophosphamide + fluorouracil	methotrexate +cyclophosphamide + fluorouracil	arrhythmia	1	!	+	+	+	+	!
	6	Pignata2011	pegylated liposomal doxorubicin	pegylated liposomal doxorubicin+carboplatin	arrhythmia	1	+	+	+	+	+	+

+ Low risk
! Some concerns
- High risk

D1 Randomisation process
D2 Deviations from the intended interventions
D3 Missing outcome data
D4 Measurement of the outcome
D5 Selection of the reported result

	Randomization process	Deviations from intended interventions	Mising outcome data	Measurement of the outcome	Selection of the reported result	
Assignment to intervention (the 'intention-to-treat' effect)						
Total number of study = 2						
Low risk	50	50	100	100	50	50
Some concerns	50	50	0	0	50	50
High risk	0	0	0	0	0	0
Adhering to intervention (the 'per-protocol' effect)						
Total number of study = 6						
Low risk	33.3	83.3	100	83.3	100	33.3
Some concerns	66.7	16.7	0	16.7	0	66.7
High risk	0	0	0	0	0	0

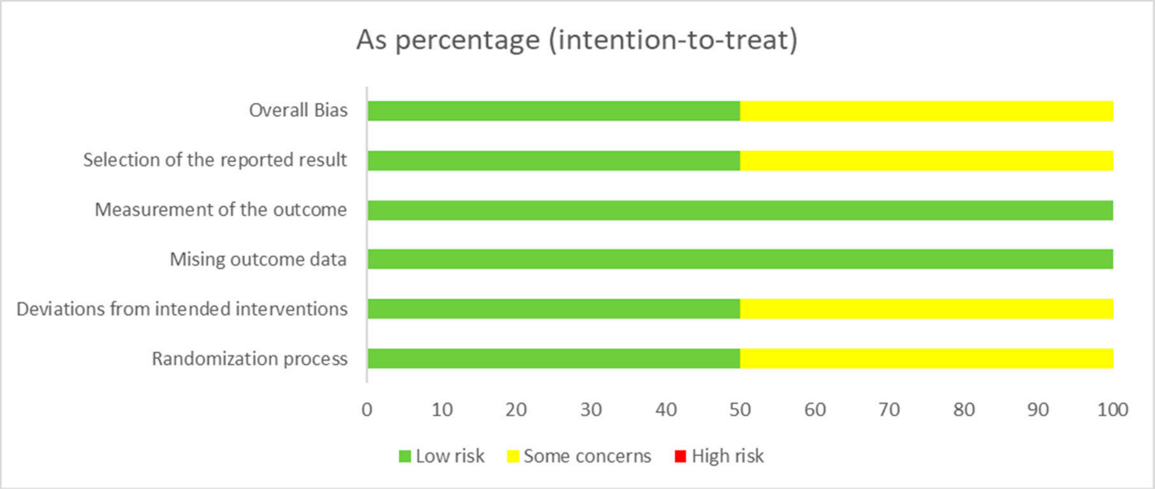
As percentage (intention-to-treat)
Overall Bias
Selection of the reported result
Measurement of the outcome
Mising outcome data
Deviations from intended interventions
Randomization process
0
10
20
30
40
50
60
70
80
90
100
Low risk
Some concerns
High risk

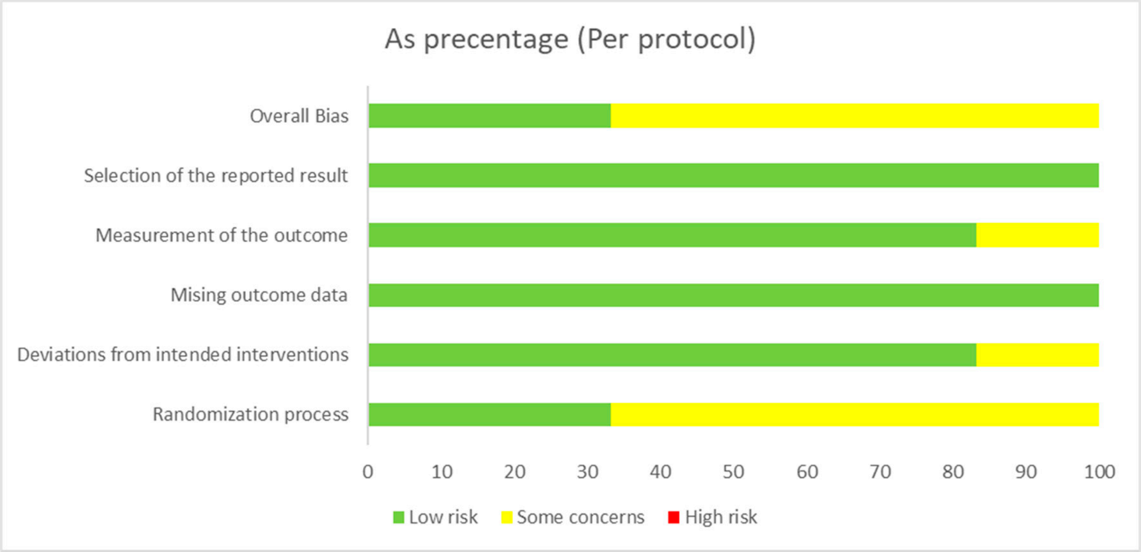
As precentage (Per protocol)
Overall Bias
Selection of the reported result
Measurement of the outcome
Mising outcome data
Deviations from intended interventions
Randomization process
0
10
20
30
40
50
60
70
80
90
100
Low risk
Some concerns
High risk

Author(s): Hongzheng Li*, Wenwen Yang*, Jingjing Wang, Zikai Yu, Mingyan Huang, Yuxuan Peng, Ling Tan, Aimei Lu, Feifei Liao, Mi Deng, Linzi Long, Hua Qu, Changgeng Fu

Date: 2022-06-06

Question: Should anthracyclines vs non-anthracyclines be used in patients with cancer?

Settings: arrhythmias

Bibliography: Risk of Arrhythmia with Exposure to Anthracyclines: A Systematic Review, Meta-Analysis, and Network Meta-Analysis

Quality assessment							No of patients		Effect		Quality	Importance
**No of studies**	**Design**	**Risk of bias**	**Inconsistency**	**Indirectness**	**Imprecision**	**Other considerations**	**Anthracyclines**	**Non-anthracyclines**	**Relative (95% CI)**	**Absolute**		
**Risk of arrhythmia (follow-up 6-96 months; measured with: electrocardiography; range of scores: 1-260; Better indicated by lower values)**												
9	observational studies[1,1]	serious[2]	no serious inconsistency	no serious indirectness	serious[3]	dose response gradient[4]	4907	4994	-	OR 1.90 higher (1.62 to 2.24 higher)	⊕OOO VERY LOW	CRITICAL
**Risk of supraventricular arrhythmia (follow-up 6-72 months; measured with: electrocardiography; range of scores: 1-152; Better indicated by lower values)**												
3	observational studies[5]	serious[6]	no serious inconsistency	no serious indirectness	serious[3]	dose response gradient[4]	2339	3221	-	OR 2.14 higher (1.18 to 3.89 higher)	⊕OOO VERY LOW	IMPORTANT

[1] three cohort studies and six RCTs

[2] Three cohort studies lack of blinding.

[3] Total number of arrhythmia is less than 300.

[4] There exists dose-response gradient in anthracyclines.

[5] one cohort study and two RCTs.

[6] One cohort study lack of blinding.
